# Feasibility and Adoption of Low-Volume Ultrasound-Guided Superior Trunk Block for Shoulder Reduction in the Emergency Department

**DOI:** 10.3390/jcm15166245

**Published:** 2026-08-12

**Authors:** Eckehart Schöll, Werner Vach, Dirk Maier, Andreas Marc Müller, Rainer Jürgen Litz

**Affiliations:** 1 Emergency Department, Bethesda Spital, 4052 Basel, Switzerland; 2 Basel Academy for Quality and Research in Medicine, 4051 Basel, Switzerland; 3 Department of Orthopedics and Traumatology, University Hospital, 4031 Basel, Switzerland; 4 Independent Researcher, 86199 Augsburg, Germany

**Keywords:** shoulder dislocation, ultrasound-guided regional anesthesia, superior trunk block, emergency department, procedural sedation, implementation

## Abstract

**Background**: Procedural sedation and analgesia (PSA) is commonly used for shoulder dislocation reduction in emergency departments (EDs), but it requires monitoring resources and may be associated with sedation-related adverse events. Ultrasound-guided regional anesthesia (UGRA), particularly low-volume superior trunk (ST) block, has emerged as a potential alternative. However, little is known about how this technically demanding technique can be implemented and adopted in routine ED practice. This study aimed to describe the implementation and clinical adoption of low-volume ultrasound-guided ST block for shoulder reduction in a specialized ED over a six-year period. **Methods**: This retrospective single-center observational cohort study included all consecutive patients undergoing shoulder reduction in a specialized orthopedic ED between February 2018 and February 2024. Patients were managed according to routine clinical practice using either UGRA or PSA, with treatment choice determined by the treating physician. The primary objective was to describe the implementation and clinical adoption of low-volume ST block in routine practice. Additional analyses assessed temporal trends in technique utilization, local anesthetic volume, provider distribution, and ED length of stay. **Results**: A total of 206 patients were included (124 UGRA; 82 PSA). The use of UGRA increased progressively during the study period, whereas PSA decreased accordingly (*p* < 0.001). Most UGRA procedures were performed by a small group of experienced physicians, reflecting the gradual adoption of the technique in routine clinical practice. Local anesthetic volumes decreased significantly over time (*p* < 0.001), with most blocks ultimately performed using approximately 4–5 mL. Successful shoulder reduction was achieved in all patients. No conversion from UGRA to PSA was necessary. No clinically relevant complications such as respiratory or hemodynamic distress were observed in either group, although diaphragmatic function was not systematically assessed. **Conclusions**: This retrospective implementation analysis demonstrates that low-volume ultrasound-guided ST block was increasingly integrated into routine ED practice. The findings primarily describe the implementation and progressive adoption of a technically demanding regional anesthesia technique in a real-world setting. Prospective studies are warranted to further evaluate clinical effectiveness, patient-reported outcomes, and respiratory effects using standardized study protocols.

## 1. Introduction

Acute shoulder dislocation (SD) is a common and painful condition frequently encountered in emergency departments (EDs), with an incidence of approximately 25–35 cases per 100,000 persons per year [1,2].

Procedural sedation and analgesia (PSA) is widely used to facilitate reduction [3], providing analgesia and reducing muscle strain. However, PSA may be associated with potential adverse events such as respiratory depression, hemodynamic instability, and prolonged ED stay [4]. Hence, PSA requires continuous patient monitoring and dedicated personnel during both the procedure and recovery phase, which may increase staff workload and impact resource availability in busy ED settings [5,6].

Ultrasound-guided regional anesthesia (UGRA) has emerged as an alternative approach for shoulder reduction [7,8,9,10,11]. In particular, interscalene brachial plexus block (ISB), which is commonly used for shoulder procedures, is associated with a high incidence of hemidiaphragmatic paralysis due to phrenic nerve (PN) blockade as an inevitable consequence of its close proximity to the plexus at this level [12,13]. Even with low local anesthetic volumes and modified injection techniques, clinically relevant diaphragmatic paralysis and respiratory complications such as dyspnea cannot be reliably avoided [12,14].

More recently, targeted, more peripheral approaches such as the superior trunk (ST) block have been proposed as an alternative to classical ISB. By increasing the anatomical distance to the PN while ensuring local anesthetic (LA) spread to all nerves supplying the surgical site, this approach may reduce the likelihood of PN involvement [12,14,15]. When performed with low volumes of LA, ST block has therefore attracted increasing interest as a potentially diaphragm-sparing technique for shoulder procedures. However, evidence regarding its implementation in routine ED practice remains limited [13,16].

As opposed to surgery where dense anesthesia is required, emergency physicians must integrate effective tailored analgesic techniques into a fast-paced clinical environment characterized by time pressure, variable staffing, and heterogeneous operator experience. Consequently, successful implementation of UGRA depends not only on technical feasibility but mainly on training, supervision, procedural exposure, and organizational factors.

The present retrospective observational study describes the implementation and clinical adoption of low-volume ultrasound-guided ST block for shoulder reduction in routine ED practice. In addition, temporal changes in technique utilization, LA volume, provider distribution, and ED workflow were analyzed. Comparative analyses between treatment groups were performed as secondary analyses and, because of the retrospective observational design, should be regarded as hypothesis-generating rather than confirmatory.

## 2. Methods

### 2.1. Study Design and Setting

This was a retrospective, single-center observational implementation analysis conducted at a specialized orthopedic ED in Switzerland. The department manages patients with acute musculoskeletal conditions, including SDs, in a clinical environment characterized by rotating staff with variable procedural experience.

All data were derived from routine clinical care. The study period extended from February 2018 to February 2024, during which UGRA techniques were progressively introduced into clinical practice.

During the study period, a total of 31 physicians from different specialties were employed in the ED, including anesthesiologists, orthopedic surgeons, internists, general practitioners, and residents. Clinical care was provided by a rotating team with varying procedural experience and exposure to UGRA.

### 2.2. Participants

All consecutive patients presenting with acute SD during the study period were considered for analysis.

Inclusion criteria were age ≥ 15 years, radiologically confirmed SD, and treatment in the ED. Patients were included irrespective of the analgesia technique used.

Exclusion criteria were missing essential clinical data and primary surgical management without attempted closed reduction.

### 2.3. Implementation

The implementation of UGRA in the ED was supported by a structured continuous training program and supervision.

All physicians underwent basic ultrasound training at the beginning of their employment, including certified courses in ultrasound-guided interventions and supervised clinical practice. In addition, hands-on training in regional anesthesia techniques was provided during routine clinical care by experienced operators.

The introduction of the technique evolved progressively as part of routine clinical practice.

### 2.4. Interventions/Clinical Management

Patients underwent closed reduction in SD either under PSA or UGRA.

The choice of analgesia technique reflected routine clinical decision-making rather than a predefined study protocol. Treatment allocation was based on physician preference, individual expertise with UGRA, patients’ request, and organizational limitations within the ED. Consequently, treatment allocation was not randomized and potential selection bias must be considered when interpreting results.

Procedural sedation was performed according to institutional standards using commonly applied sedative and analgesic agents under continuous monitoring of vital signs. Patients were observed during both the procedure and recovery phase according to standard ED protocols.

Ultrasound-guided regional anesthesia was performed using a ST block technique as described below.

### 2.5. Ultrasound-Guided Regional Anesthesia Technique

Ultrasound-guided brachial plexus block was performed by targeting the ST in the supraclavicular region. Procedures were performed using either a Samsung RS80 EVO (Samsung Medison, Seoul, Republic of Korea) with an LA4-18B linear transducer or a Canon Aplio a (Canon Medical Systems, Tokyo, Japan) with a PLT-1204BT linear transducer (18 MHz).

**Figure 1:**
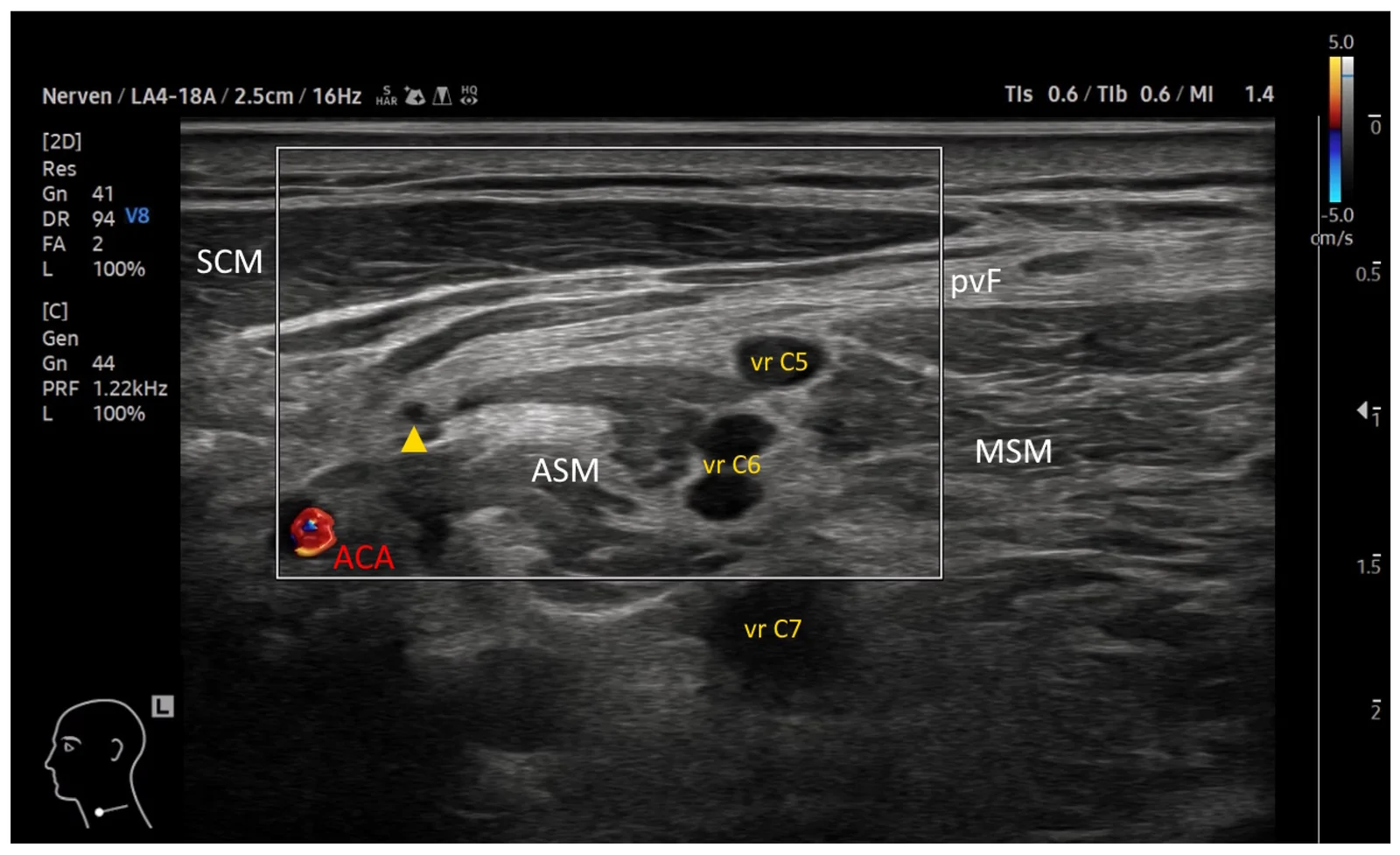
Interscalene level and proximity of the phrenic nerve. Ultrasound image obtained at the classical interscalene level demonstrating the ventral rami (vr) of C5–C7 located between the anterior scalene muscle (ASM) and middle scalene muscle (MSM). The phrenic nerve (yellow arrowhead) is visible beneath the prevertebral fascia (pvF) in close proximity to the brachial plexus within the same compartment. SCM = sternocleidomastoid muscle; ACA = ascending cervical artery.

To identify the relevant anatomy, the ultrasound probe was initially placed in the interscalene groove, where the ventral rami of C5–C7 can be visualized between the anterior and middle scalene muscles (Figure 1). At this level, the PN, emerging predominantly from C4, courses in close proximity in the same compartment beneath the prevertebral fascia.

**Figure 2:**
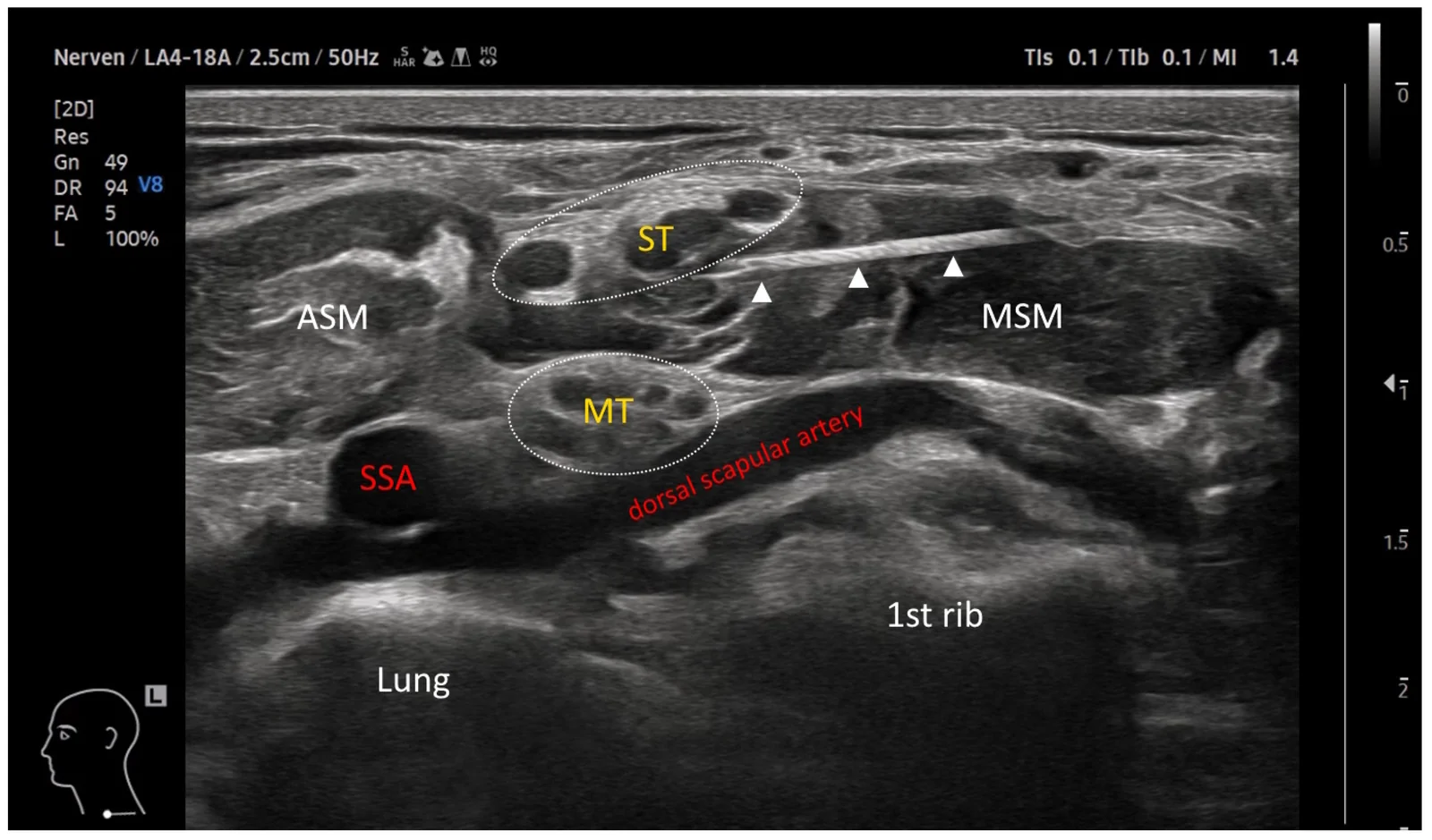
Supraclavicular approach to the superior trunk. Ultrasound image in the supraclavicular region demonstrating the superior trunk (ST) as the primary target. The middle trunk (MT), suprascapular artery (SSA), and first rib are visible. The pleura/lung is seen deep to the rib. Compared to the interscalene level, the phrenic nerve is typically located more medially and farther away from the ST. The injection needle with its visible tip is indicated by white arrows.

The probe was then shifted caudally to the supraclavicular region, where the ST becomes identifiable and the PN typically courses more medially and farther away from the ST (Figure 2).

The block was performed under real-time ultrasound guidance using an in-plane approach. After positioning the needle tip beneath the ST, LA was slowly injected under continuous visualization of its spread. If necessary, the needle tip was repositioned to ensure adequate circumferential distribution around the ST.

**Figure 3:**
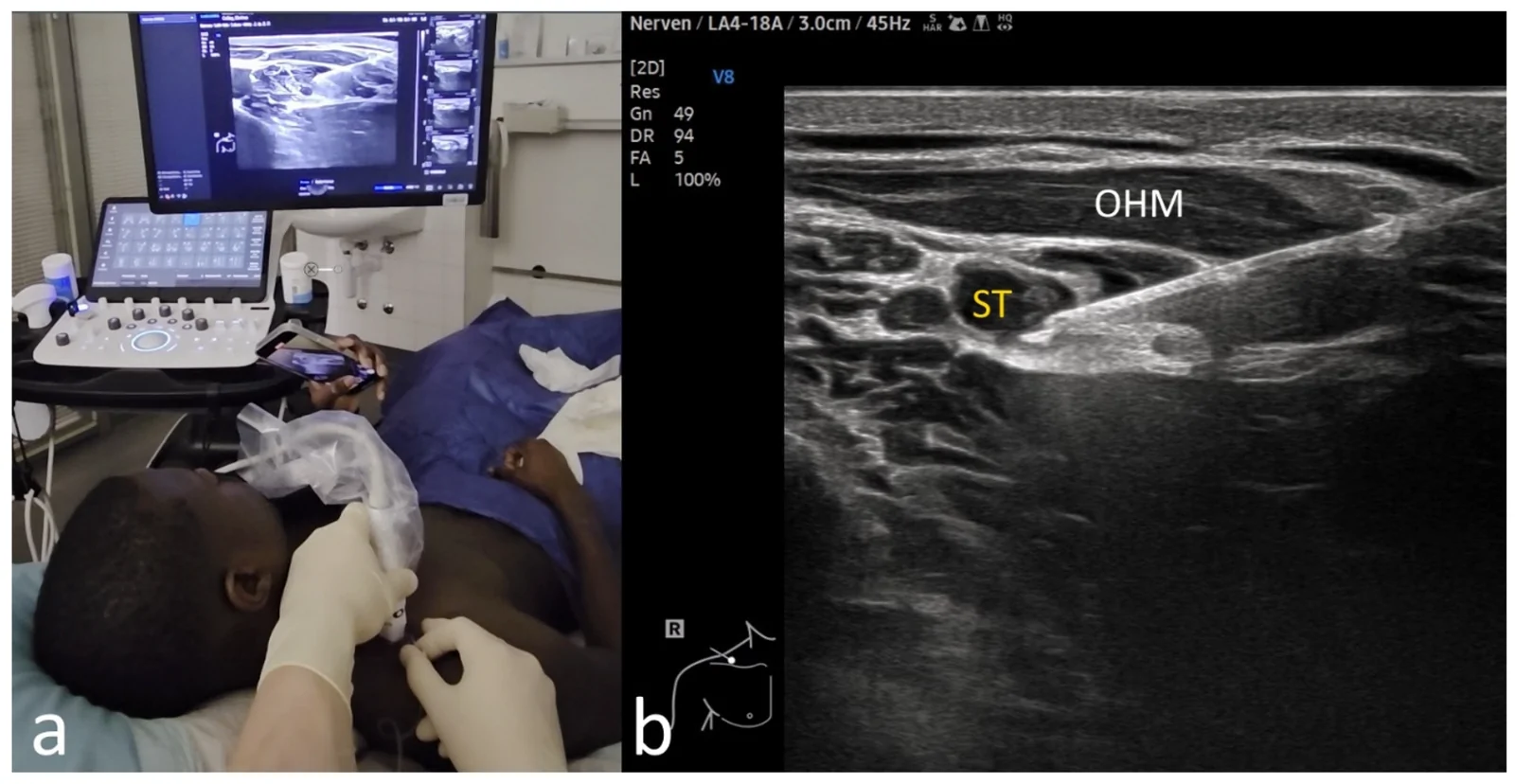
Ultrasound-guided superior trunk block technique. (**a**) Clinical setup showing the patient position and in-plane needle approach in the supraclavicular region. (**b**) Corresponding ultrasound image demonstrating the needle tip advanced beneath the superior trunk (ST) with the omohyoid muscle (OHM) as an additional anatomical landmark.

The procedure was performed under routine clinical conditions in the ED, as illustrated in Figure 3.

A LA volume of approximately 5 mL was initially used in clinical practice and adjusted according to the individual spread pattern. Neither the LA nor the injected volume was fixed, reflecting routine clinical practice rather than a predefined study protocol. Most procedures were performed using 2% prilocaine, whereas one operator consistently used 0.75% ropivacaine throughout the study period. Blocks were considered clinically sufficient if no further analgesics were required throughout the procedure.

## 3. Data Collection

Clinical data were retrospectively extracted from electronic medical records and included patient demographics, type of analgesia technique (PSA vs. UGRA), LA volume, treating physician, and length of stay (LOS) in the ED.

LOS was defined as the time from patient admission to discharge from the ED. Patients requiring subsequent surgery were excluded from LOS analyses, as discharge occurred several days later and was therefore not representative of ED stay.

## 4. Outcome Measures

The primary aim of the study was to describe the implementation process, feasibility, and clinical adoption of UGRA for shoulder reduction in routine clinical practice.

As this was a retrospective implementation analysis, feasibility was assessed using routinely documented clinical indicators, including successful completion of shoulder reduction under UGRA without conversion to an alternative analgesic technique. Accordingly, successful completion of shoulder reduction without additional analgesics was considered an indicator of clinically sufficient block performance under routine ED conditions.

Adoption in clinical practice was assessed by temporal trends in the use of UGRA over the study period. Further aspects of the adoption process were assessed by temporal trends in LA volume and LOS. The latter was also performed in PSA patients in order to control for general time trends.

Finally, the distribution of LOS was compared between UGRA and PSA patients to allow comparison with existing efficiency studies.

No predefined primary endpoint or prospective sample size calculation was performed due to the retrospective nature of the study.

## 5. Statistical Analysis

Statistical analyses were primarily descriptive.

Temporal trends were explored using linear regression models. Statistical significance of differences in the distribution of the LOS between groups was assessed using the Wilcoxon rank sum test.

A two-sided *p*-value < 0.05 was considered statistically significant.

## 6. Ethics

The study was approved by the Ethics Committee of Northwestern and Central Switzerland (EKNZ; Project ID 2025-02605; approval date: 20 April 2026). The study was post hoc registered as an observational study at ClinicalTrials.gov (NCT07544485; registration date: 21 April 2026). The requirement for informed consent was waived due to the retrospective analysis of routinely collected, anonymized clinical data.

## 7. Results

### 7.1. Study Population

**Figure 4:**
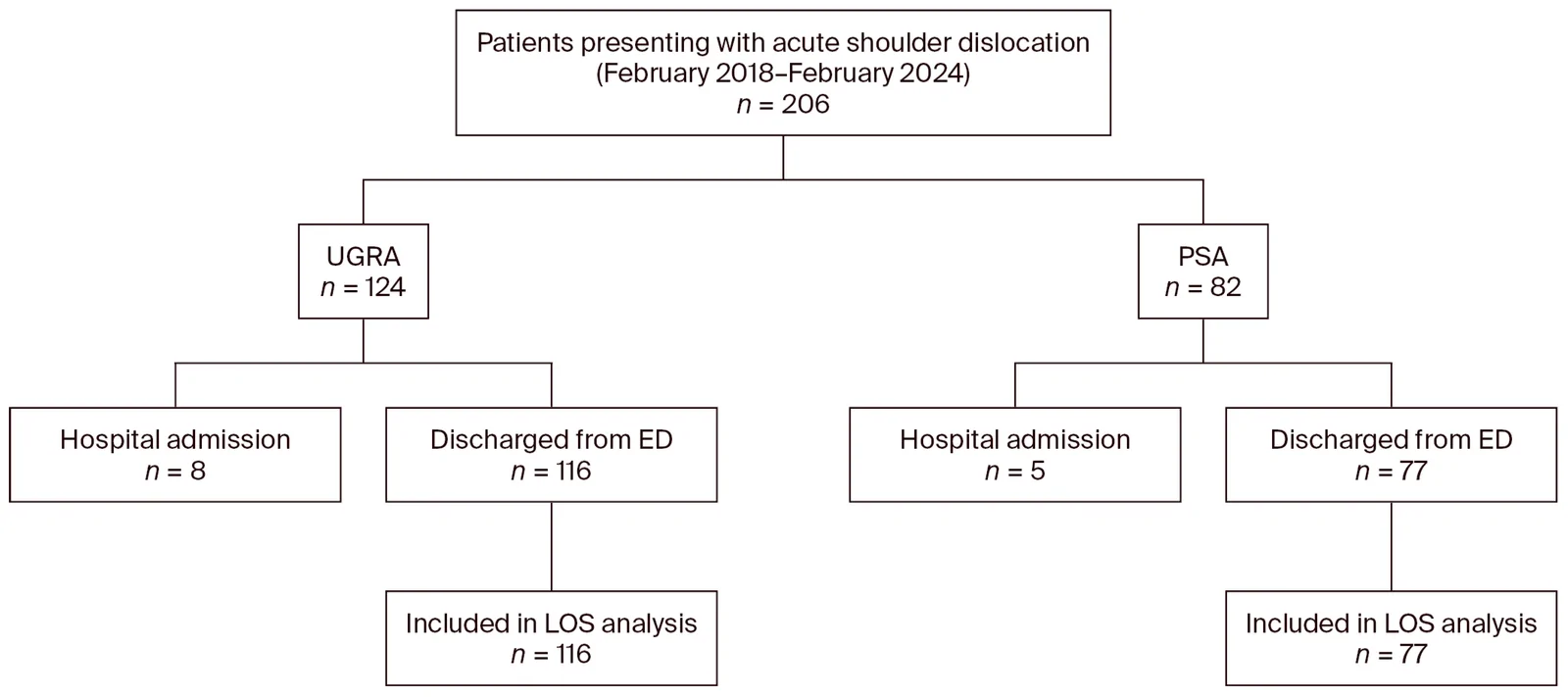
Patient selection and inclusion in exploratory length-of-stay (LOS) analysis. All patients presenting with acute shoulder dislocation during the study period were included in the primary analysis. No patients were excluded due to missing data. Patients requiring hospital admission (UGRA *n* = 8; PSA *n* = 5) were excluded from LOS analyses.

During the study period from February 2018 to February 2024, a total of 206 patients (73 female, 133 male) with a median age of 48.4 years (range 15.3–93.7) were included in the analysis. Patient selection and inclusion in the exploratory LOS analysis are summarized in Figure 4.

### 7.2. Feasibility

Closed reduction was performed under UGRA in 124 patients (60%) and under PSA in 82 patients (40%). Radiographically confirmed successful reduction was achieved in all patients without the need for conversion to alternative analgesic techniques. No major complications such as respiratory or hemodynamic distress requiring treatment were identified in either group.

**Figure 5:**
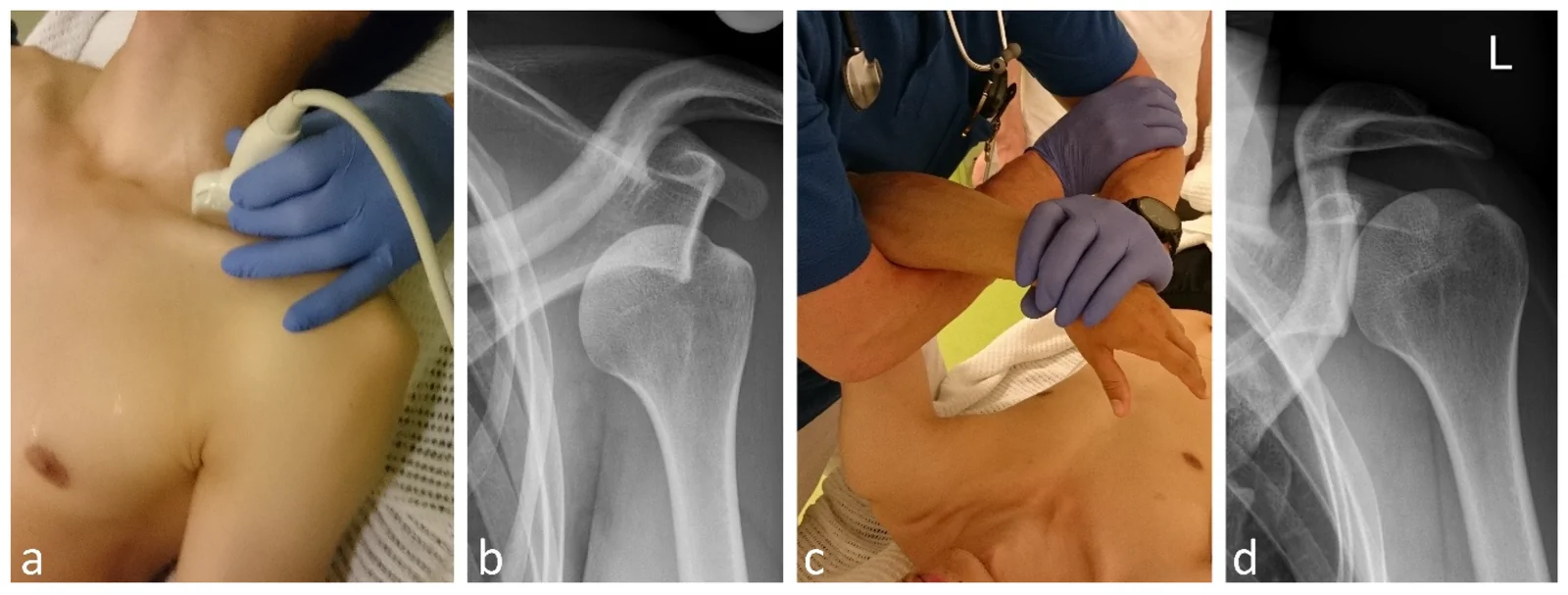
Clinical course of shoulder dislocation and reduction. (**a**) Clinical presentation of a patient with acute shoulder dislocation with ultrasound probe in the supraclavicular region. (**b**) Pre-reduction radiograph confirming anterior shoulder dislocation. (**c**) Reduction maneuver performed in the ED. (**d**) Post-reduction radiograph demonstrating successful joint re-alignment.

A representative clinical course of SD and reduction is shown in Figure 5.

### 7.3. Adoption of UGRA over Time

A total of 31 physicians were involved in patient care during the study period. However, the distribution of UGRA procedures among providers was highly heterogeneous. Four anesthesiologists performed the majority of blocks (*n* = 88), whereas the remaining 27 physicians performed only 36 blocks in total.

**Figure 6:**
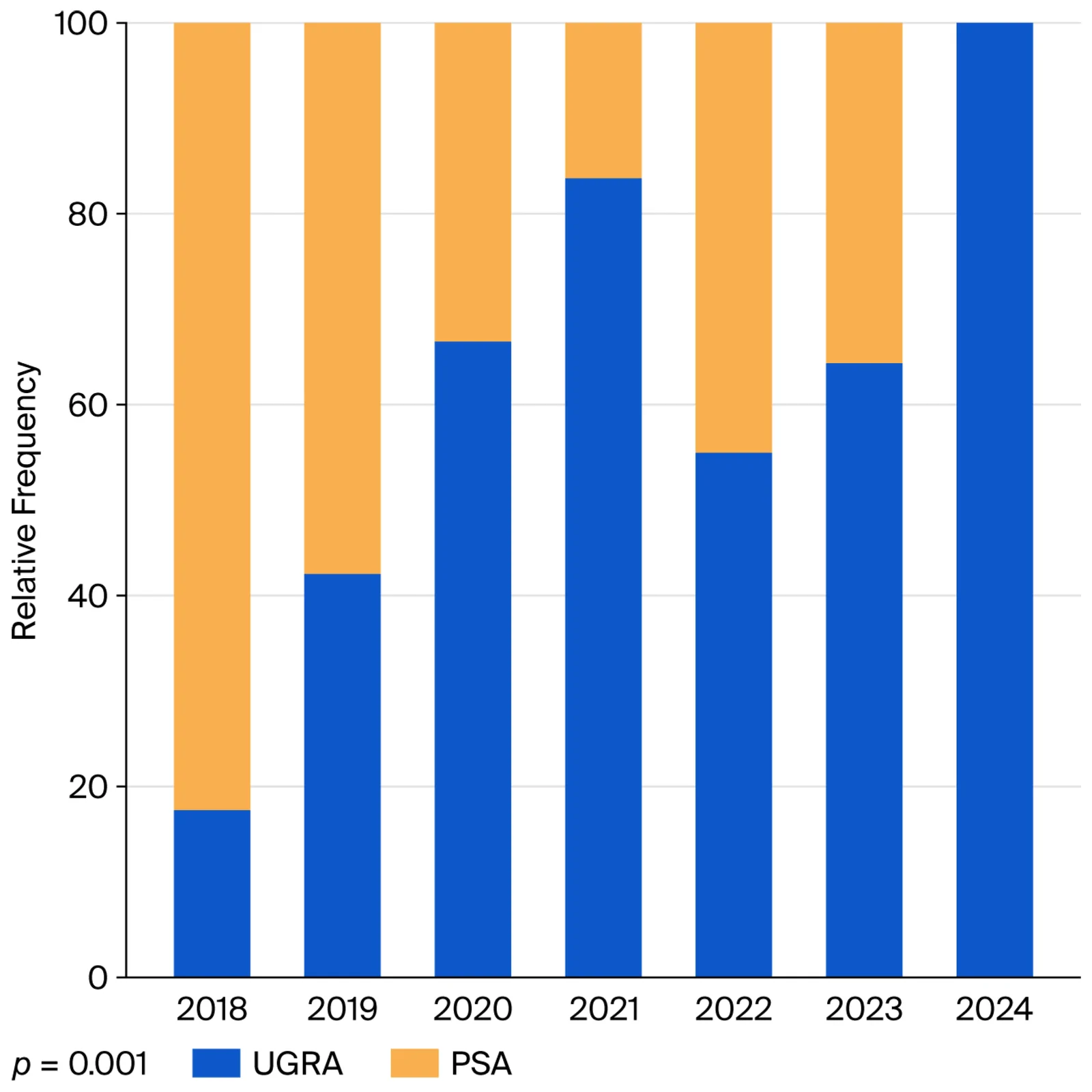
Use of analgesia techniques over time. Temporal distribution of analgesia techniques during the study period, demonstrating an increasing use of UGRA and a corresponding decrease in PSA.

Over the six-year study period, the use of UGRA increased steadily, while the proportion of PSA decreased accordingly (Figure 6). This trend was statistically significant (*p* < 0.001) and reflects the progressive adoption of UGRA in routine clinical practice.

### 7.4. Local Anesthetic Volume

**Figure 7:**
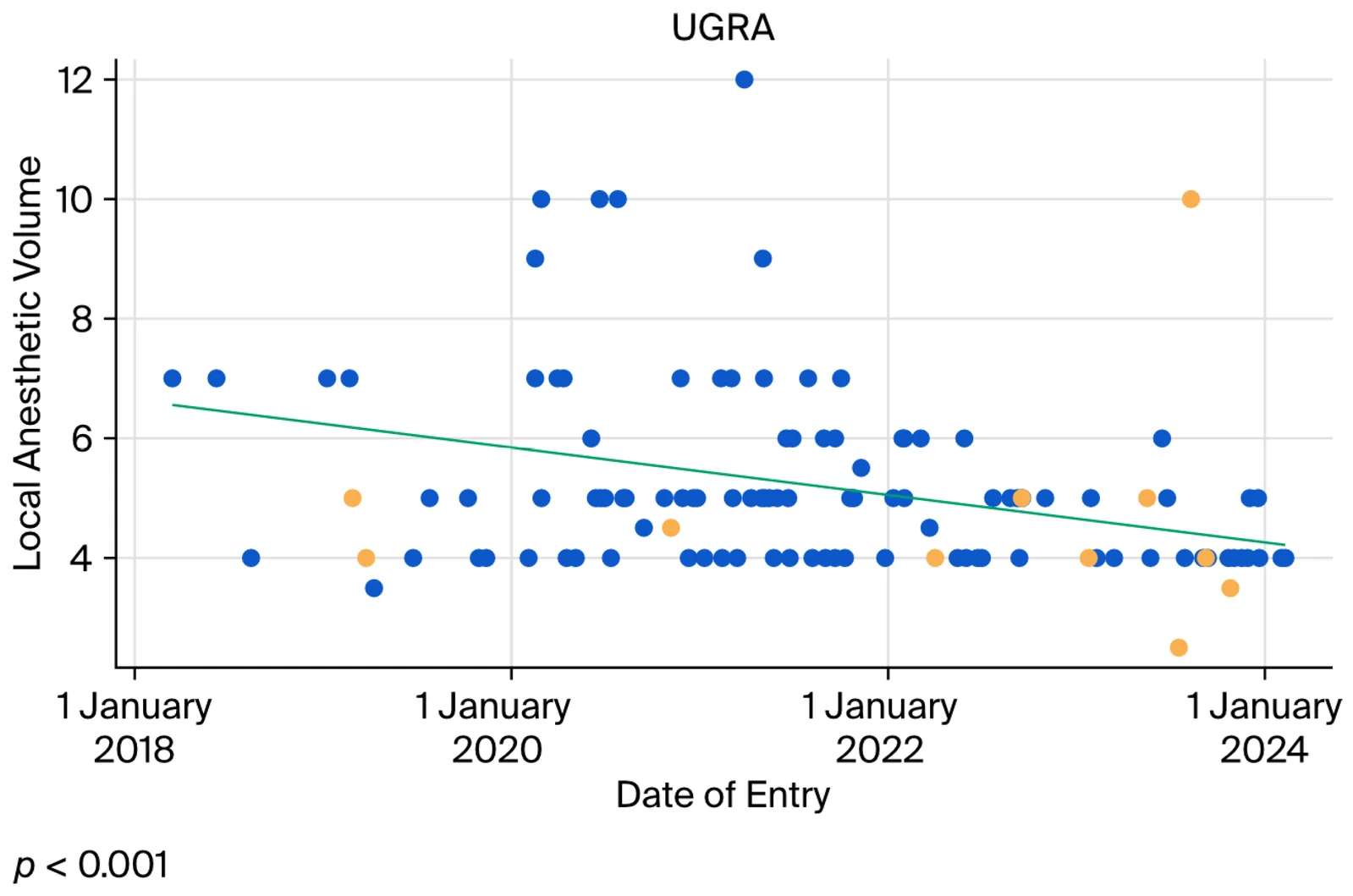
Local anesthetic volume over time. Temporal trend in LA volume administered during ultrasound-guided blocks, demonstrating a progressive reduction over the study period. Blue dots = prilocaine, yellow dots = ropivacaine. The green line represents the fitted linear regression line.

The volume of LA decreased over time (Figure 7). Analysis of all blocks demonstrated a significant reduction in administered volumes during the study period (*p* < 0.001).

Most procedures were performed using volumes of approximately 4–5 mL. Due to the limited number of procedures per individual provider, operator-specific analyses were not further explored in the present study.

### 7.5. Temporal Trends in LOS

Length of stay (LOS) was analyzed in patients discharged from the ED. Patients requiring hospital admission were excluded (8 UGRA, 5 PSA).

**Figure 8:**
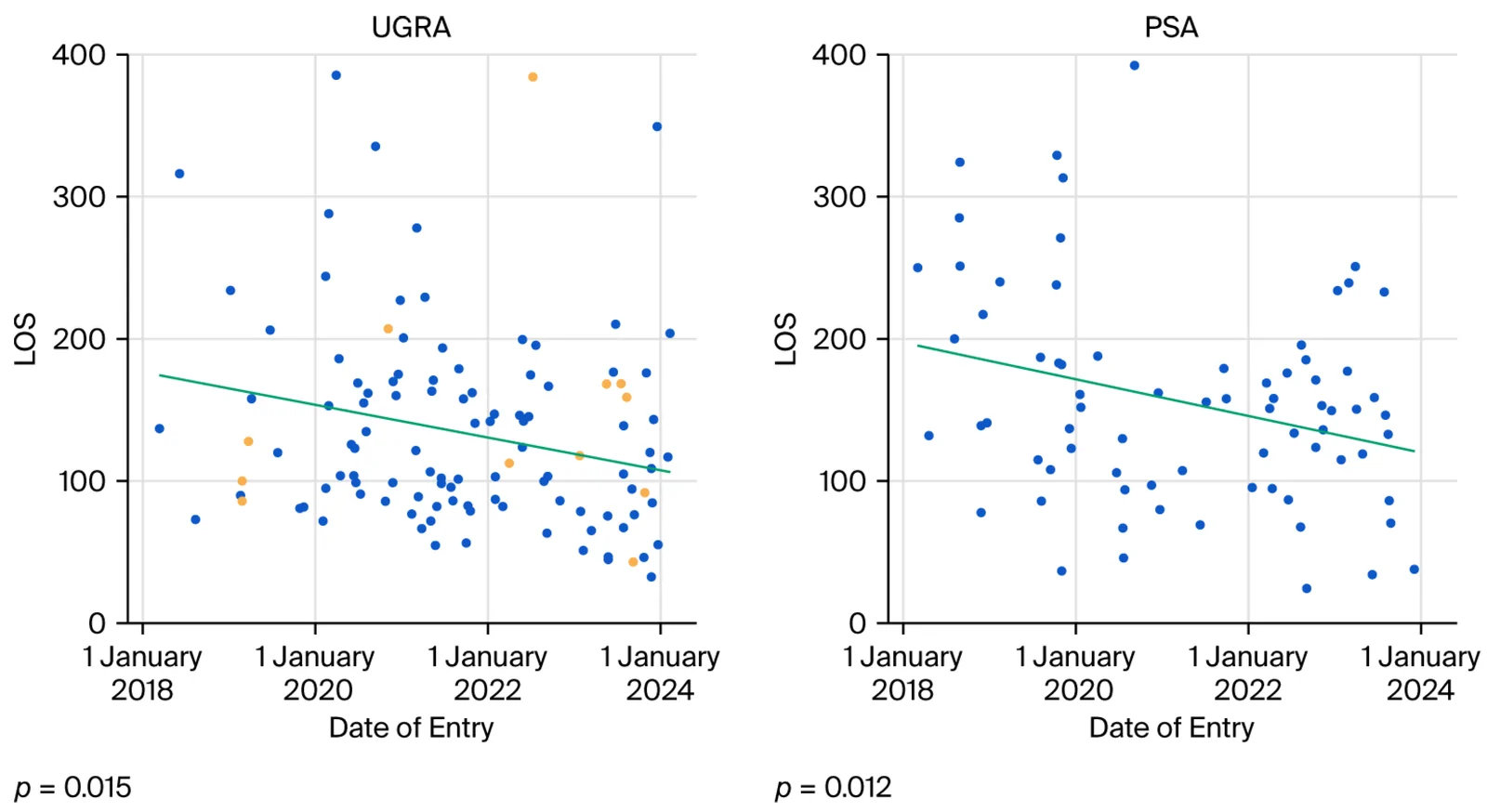
Temporal trends in length of stay. Temporal changes in LOS for patients treated with UGRA and PSA. A reduction in LOS over time was observed in both treatment groups. UGRA-Group: Blue dots = prilocaine, yellow dots = ropivacaine. The green lines represent the fitted linear regression lines.

A reduction in LOS over time was observed in both treatment groups (Figure 8). Linear trend analyses demonstrated decreasing LOS for both UGRA and PSA during the study period (UGRA: *p* = 0.015; PSA: *p* = 0.012), suggesting that general improvements in departmental workflow over time may also have contributed to shorter treatment times.

**Figure 9:**
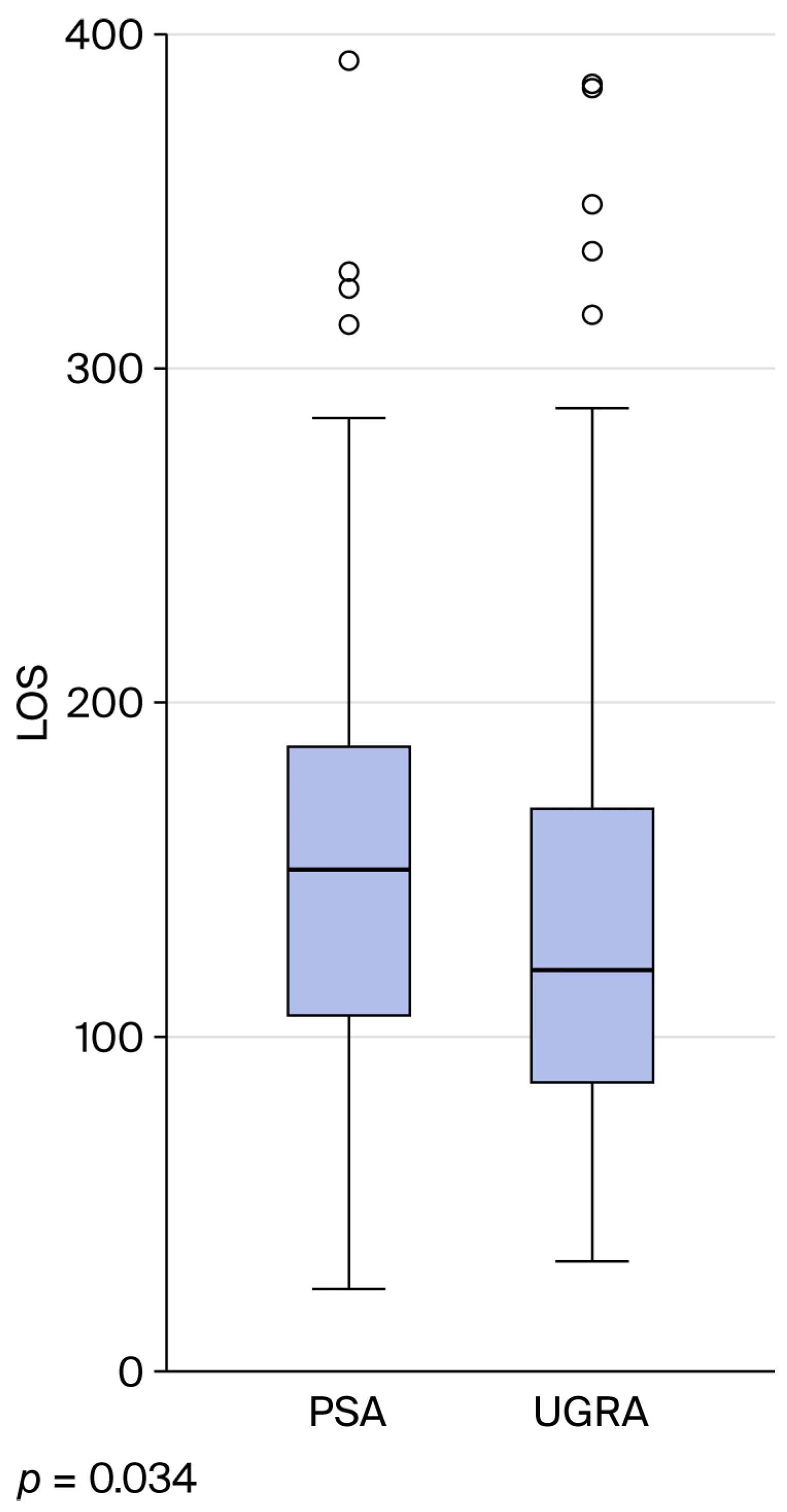
Length of stay comparison. Comparison of LOS between patients treated with UGRA and PSA. A shorter LOS was observed in the UGRA group.

Patients treated with UGRA showed a shorter median LOS compared to those treated with PSA, with an approximate difference of 30 min (*p* = 0.034; Figure 9). Variability in LOS was considerable.

## 8. Discussion

This retrospective implementation analysis demonstrates that ultrasound-guided low-volume ST block can be successfully integrated into routine ED practice. Rather than evaluating comparative clinical effectiveness, the present study primarily describes the adoption of a technically demanding UGRA technique under real-world conditions.

Our observations are consistent with a recently published case series reporting successful shoulder reductions using low-volume ST blocks [17]. Although that report included only a small number of patients, it likewise supports the feasibility of performing shoulder reduction under low-volume ST block. In contrast to the present implementation analysis, however, the published case series primarily focused on procedural feasibility rather than the longitudinal adoption of the technique in routine ED practice.

Previous studies have demonstrated that UGRA is an effective alternative to PSA for shoulder reduction, primarily focusing on analgesic efficacy, procedural success, and short-term clinical outcomes [7,8,9,10,11]. In contrast, little attention has been paid to the practical integration and long-term adoption of these techniques in routine ED practice. The present study therefore complements the existing literature by focusing on implementation rather than comparative effectiveness.

A key finding of the present study was the progressive increase in the use of UGRA over time, corresponding with a decrease in PSA. This trend, which was statistically significant, suggests growing acceptance of the technique among clinicians and its gradual integration into routine clinical practice. Similar patterns of increasing uptake following structured implementation efforts have been described in other ED settings, although overall utilization and adoption across providers remain highly variable depending on training and local practice conditions [18,19,20].

Despite this overall trend, the distribution of procedural experience among providers was highly heterogeneous. In the present study, four anesthesiologists performed the majority of UGRA procedures, while the remaining physicians had only limited exposure. This finding highlights a key challenge in the integration of technically demanding procedures in ED settings.

The limited number of cases per individual provider, combined with high staff turnover, likely restricted the opportunity for many physicians to acquire and maintain sufficient procedural competence. As a result, even in a high-volume center, the number of procedures may not be sufficient to ensure widespread proficiency across all providers. This challenge is likely not unique to ST block but reflects a general limitation of implementing low-frequency procedural skills in emergency medicine, where individual procedural exposure may be insufficient for many physicians. Sustainable implementation therefore depends not only on the availability of the technique itself but also on structured teaching, sufficient procedural exposure, continuous supervision, and institutional support to maintain competence over time. Similar barriers to implementation have been described in previous emergency medicine studies [19,20]. Nevertheless, throughout the study period no conversion from UGRA to PSA or general anesthesia was necessary in any patient, suggesting that the block was considered clinically sufficient to allow successful shoulder reduction under routine ED conditions.

One potential strategy to overcome this limitation may be the broader application of UGRA techniques to other shoulder-related conditions in emergency settings, thereby increasing overall procedural exposure.

Ultrasound-guided regional anesthesia has been extensively described for a variety of shoulder pathologies beyond SD, including adhesive capsulitis, rotator cuff disease, and chronic shoulder pain, particularly using suprascapular blocks and ISB. Several randomized and observational studies have also demonstrated effective analgesia and functional improvement in these conditions [21,22,23]. These findings support the concept that broader clinical application may facilitate skill acquisition, improve procedural confidence, and enhance the integration of regional anesthesia techniques into routine emergency care.

A progressive reduction in LA volume was observed during the implementation process. This finding likely reflects increasing operator confidence and more precise ultrasound-guided needle placement as procedural experience accumulated. This observed reduction should be interpreted as a descriptive finding rather than evidence of an optimal injection volume. The latter would require a dedicated dose finding study. Reduced LA volumes have been shown to decrease the risk of PN involvement without compromising block efficacy [11,12]. In this context, low-volume approaches may represent a clinically relevant strategy when performing ST blocks.

Comparative studies between interscalene and ST blocks suggest that targeting the ST reduces the incidence of PN involvement even when higher LA volumes (15–20 mL) are used, highlighting the anatomical advantage of this approach [14,15].

In our cohort, no clinically apparent respiratory compromises were observed following UGRA. Because diaphragmatic function was not systematically evaluated by ultrasound or pulmonary function testing, no conclusions regarding the true incidence of PN involvement or significant hemidiaphragmatic paresis can be drawn. Accordingly, the present findings should not be interpreted as evidence that low-volume ST block always prevents hemidiaphragmatic paresis. Moreover, because this was a retrospective study based on routine clinical documentation, the occurrence of any minor adverse effects or complications cannot be excluded. The absence of clinically relevant respiratory symptoms in our ED may be related to the combination of the more distal injection site and the slow injection of low LA volumes (approximately 5 mL) in our cohort.

Similarly, neurological injury is a recognized and primarily trauma-related complication of anterior SD, with reported incidences ranging from 0.4% to 65.5%, depending largely on the diagnostic methodology used. The axillary nerve is the most frequently affected nerve [24]. Most of these nerve injuries resolve spontaneously. In our study, standardized neurological follow-up after reduction was not available for two reasons. First, the study was designed as a retrospective implementation analysis rather than an outcome study. Second, due to the short ED stay in many patients there was a residual local anesthetic effect at discharge. Consequently, transient or persistent axillary nerve deficits were not evaluated systematically in the ED and were subsequently evaluated during orthopedic follow-up. Data from subsequent outpatient follow-up were not available in our study and should be addressed in future prospective investigations.

In previous comparative studies, UGRA has been associated with shorter treatment times and more efficient patient throughput [8,11]. A similar finding was observed in the present study: Patients treated with UGRA showed a shorter LOS compared to those undergoing PSA. However, this cannot be regarded as an independent confirmation, as the retrospective design does not allow for control of confounding factors. The limitation in interpreting this finding is also illustrated by observing a reduction in LOS over time in both treatment groups. The difference in LOS between the groups was likely related to the avoidance of sedation-related monitoring and recovery phases, which may have contributed to improved workflow efficiency in the ED, as PSA typically requires continuous monitoring and dedicated personnel resources in accordance with current guidelines [6].

From a clinical perspective, ultrasound-guided low-volume ST block represents a practical addition to the available analgesic strategies for shoulder reduction in the ED. Rather than replacing PSA, it may serve as an effective complementary approach in most patients, particularly when systemic sedation should be avoided or resource availability is limited.

## 9. Limitations

The findings of this retrospective implementation analysis should be interpreted in the light of several limitations.

First, treatment allocation reflected routine clinical decision-making rather than protocol-driven assignment and may change over time. Consequently, time trends in outcomes and group differences can be affected by potential selection bias and unmeasured confounding, precluding causal interpretations.

Second, given the retrospective exploratory design, patient-relevant outcomes such as pain scores before and after block placement, procedural discomfort, patient-reported experiences, and diaphragmatic function were not systematically documented and therefore could not be analyzed.

Third, operator experience varied considerably, and individual learning curves could not be reliably assessed due to limited case numbers per provider. Furthermore, most UGRA procedures were performed by a small group of experienced physicians. Therefore, the observed implementation process and clinical outcomes may not be directly generalizable to centers with different levels of expertise or procedural exposure.

Furthermore, standardized neurological outcomes after reduction were not systematically documented. Therefore, the incidence of transient or persistent axillary nerve injury could not be assessed.

Finally, this was a single-center study, which may limit generalizability.

## 10. Conclusions

Ultrasound-guided superior trunk block using a low-volume approach for the reduction in shoulder dislocation was successfully implemented in our emergency department. It provides an example of how such a technique may be implemented in a specialized emergency care setting. Its adoption requires time, training, and repeated clinical exposure, particularly in environments with high staff turnover.

The present study primarily provides a practical framework for implementing low-volume ST block in routine emergency care rather than evidence of comparative clinical superiority. Prospective studies should evaluate respiratory function, procedural conditions, patient-reported outcomes, and comparative clinical effectiveness.

## Data Availability

The data presented in this study are available on request from the corresponding author. The data are not publicly available due to patient privacy considerations and institutional data protection regulations.
